# What is the value of regional cerebral saturation in post-cardiac arrest patients? A prospective observational study

**DOI:** 10.1186/s13054-016-1509-9

**Published:** 2016-10-13

**Authors:** Cornelia Genbrugge, Ward Eertmans, Ingrid Meex, Margaretha Van Kerrebroeck, Noami Daems, An Creemers, Frank Jans, Willem Boer, Jo Dens, Cathy De Deyne

**Affiliations:** 1 Faculty of Medicine and Life Sciences, Hasselt University, Martelarenlaan 42, 3500 Hasselt, Belgium; 2 Department of Anesthesiology, Intensive Care, Emergency Medicine and Pain Therapy, Ziekenhuis Oost-Limburg Genk, Schiepse Bos 6, 3600 Genk, Belgium; 3 I-Biostat (CenStat), Hasselt University, Agoralaan gebouw D, 3590 Diepenbeek, Belgium; 4 Department of Cardiology, Ziekenhuis Oost-Limburg Genk, Schiepse Bos 6, 3600 Genk, Belgium

**Keywords:** Cerebral saturation, Post-cardiac arrest, Neurological outcome, Neuromonitoring, Targeted temperature management

## Abstract

**Background:**

The aim of this study was to elucidate the possible role of cerebral saturation monitoring in the post-cardiac arrest setting.

**Methods:**

Cerebral tissue saturation (SctO_2_) was measured in 107 successfully resuscitated out-of-hospital cardiac arrest patients for 48 hours between 2011 and 2015. All patients were treated with targeted temperature management, 24 hours at 33 °C and rewarming at 0.3 °C per hour. A threshold analysis was performed as well as a linear mixed models analysis for continuous SctO_2_ data to compare the relation between SctO_2_ and favorable (cerebral performance category (CPC) 1–2) and unfavorable outcome (CPC 3–4–5) at 180 days post-cardiac arrest in OHCA patients.

**Results:**

Of the 107 patients, 50 (47 %) had a favorable neurological outcome at 180 days post-cardiac arrest. Mean SctO_2_ over 48 hours was 68 % ± 4 in patients with a favorable outcome compared to 66 % ± 5 for patients with an unfavorable outcome (*p* = 0.035). No reliable SctO_2_ threshold was able to predict favorable neurological outcome. A significant different course of SctO_2_ was observed, represented by a logarithmic and linear course of SctO_2_ in patients with favorable outcome and unfavorable outcome, respectively (*p* < 0.001). During the rewarming phase, significant higher SctO_2_ values were observed in patients with a favorable neurological outcome (*p* = 0.046).

**Conclusions:**

This study represents the largest post-resuscitation cohort evaluated using NIRS technology, including a sizeable cohort of balloon-assisted patients. Although a significant difference was observed in the overall course of SctO_2_ between OHCA patients with a favorable and unfavorable outcome, the margin was too small to likely represent functional outcome differentiation based on SctO_2_ alone. As such, these results given such methodology as performed in this study suggest that NIRS is insufficient by itself to serve in outcome prognostication, but there may remain benefit when incorporated into a multi-neuromonitoring bedside assessment algorithm.

## Background

During a cardiac arrest (CA), the brain is exposed to hypoxia resulting in neurological injury and determining survival in the majority of the post-CA patients. The brain is namely a highly aerobic organ with a limited capacity to store energy, necessitating a constant delivery of oxygen and glucose. Regardless of the recent advances in cardiopulmonary resuscitation and post-resuscitation care, neurological injury still remains a major problem. Almost 70 % of patients who die during their hospital stay after out-of-hospital cardiac arrest (OHCA) decease due to post-anoxic neurological injury [1]. This may be explained by the fact that the brain of an OHCA patient is subjected to a sequence of pathophysiological changes during the arrest itself, but also during the return of spontaneous circulation (ROSC) and in the post-resuscitation phase. First, global ischemia of the brain occurs during the arrest which accounts for the primary neurological injury. Next, after ROSC is achieved a post-CA syndrome develops, which is characterized by a short-lasting cerebral hyperemia followed by an increase in cerebrovascular resistance finally resulting in a decrease in cerebral blood flow (CBF) [2]. During this post-CA phase, there is an imbalance between oxygen delivery relative to oxygen requirements, which can last for several hours to days. These pathophysiological changes may cause progressive and irreversible brain injury responsible for the so-called secondary neurological injury. Thus far, targeted temperature management (TTM) is the only treatment with proven efficacy on neurological outcome after OHCA [3, 4].

Current brain monitoring techniques applied in post-CA patients focus on the prediction of cerebral outcome rather than on possible therapeutic implications [5]. Hence, cerebral hemodynamics could have an influence on outcome in the post-CA phase. Therefore, a better understanding of cerebral hemodynamic disturbances via cerebral monitoring could have an impact on the post-CA management. Near-infrared spectroscopy (NIRS) provides information on brain oxygenation by monitoring the regional cerebral oxygen saturation (SctO_2_) at the microvascular level. It is a non-invasive monitoring tool to measure the difference between oxygenated and deoxygenated hemoglobin in venous, arterial, and capillary blood.

The aim of this study was to improve our knowledge and to elucidate the possible role of non-invasive SctO_2_ during the first 48 hours after an OHCA (with use of TTM at 33 °C) and to assess its possible relationship to outcome.

## Methods

### Study population

All comatose survivors after OHCA with presumed cardiac origin treated in our tertiary care hospital (Ziekenhuis Oost-Limburg, Genk, Belgium) were prospectively enrolled between March 2011 and May 2015 (*n* = 107). Exclusion criteria were patients < 18 years and an obviously non-cardiac cause of OHCA. If a fall was mentioned in the hetero-anamnesis, or if any clinical signs of a fall were present (e.g., bruises) a computed tomography (CT) scan was performed prior to coronary care unit (CCU) admission. If no clear cause of the arrest was determined at arrival at the emergency department, a head CT scan was performed to exclude cerebral causes of CA. None of the included patients had intracerebral pathologies. All patients were treated uniformly according to the institutional post-CA protocol [6]. As part of this protocol, SctO_2_ monitoring was routinely applied on arrival at the CCU. The study protocol was approved by the local medical ethics committee (Comité Medische Ethiek Ziekenhuis Oost-Limburg 11/066). Written informed consent was obtained from next of kin.

### General management

Our institutional post-CA protocol has been described previously [6]. In summary, all patients were intubated, mechanically ventilated, and sedated with propofol and remifentanil (if hemodynamically tolerated). Cisatracurium was administered in case of shivering (bolus or continuous infusion). Patients underwent urgent coronary angiography followed by percutaneous coronary intervention when indicated. TTM at 33 °C was induced as soon as possible after hospital admission by cold saline (4 °C – 30 ml/kg) and was further mechanically induced and maintained in the CCU by endovascular (Icy™ catheter, CoolGard® 3000, Alsius, Irvine, CA, USA) or surface (ArcticGel™ pads, Arctic Sun® 5000, Medivance, Louisville, CO, USA) cooling systems at 33 °C for 24 hours. Both systems are equipped with a feedback loop controlling target temperature using an esophageal temperature probe. Esophageal temperature was recorded every minute during hypothermia and rewarming. After rewarming (0.3 °C/hour for 12 hours) sedation was titrated toward patient’s comfort with efforts to minimize sedation. Patients were extubated when their neurological, respiratory and hemodynamic status had recovered sufficiently. During the first 48 hours post-CA, an hourly blood gas was taken.

### Cerebral saturation monitoring

Cerebral tissue oxygen saturation was continuously measured with NIRS, using the FORE-SIGHT™ technology (CAS Medical Systems, Branford, CT, USA). Sensors were bilaterally applied to the frontotemporal area at CCU admission, before the start of mechanically induced hypothermia. Data were transmitted to a personal computer together with all hemodynamic data with a 2-second time interval. We also calculated the area below a preset SctO_2_ threshold. This value encompasses both duration and severity of a desaturation below a preset SctO_2_ threshold during the first 48 hours after CA. Since this was an observational study, treatment was guided according to the guidelines of the European Resuscitation Council and was not affected in any way by the collected NIRS data although the SctO_2_ data were not blinded for the treating physician [7].

### Hemodynamic monitoring and management

Patients were treated according to the guidelines with the main focus on achieving a mean arterial pressure (MAP) above 65 mmHg [8]. If signs of inadequate circulation persisted despite correct fluid resuscitation (wedge pressure >18 mmHg), norepinephrine was infused first with a target MAP of 65 mmHg and subsequently dobutamine was given with a target cardiac index of >2.2 l/min/m^2^. An intra-aortic balloon pump (IABP) was installed as deemed necessary by the treating physicians. Blood pressures were obtained by a radial artery line while a Swan-Ganz catheter provided information about cardiac output (CO), cardiac index and continuous mixed venous blood oxygen saturation (SvO_2_).

### Outcome measurement

The cerebral performance category (CPC) scale was used to define patient outcome [9–11]. According to the scale classification, CPC 1 indicates good cerebral performance, CPC 2 implies a moderate disability (sufficient for independent activities in daily life), CPC 3 indicates severe disability (dependent on others), CPC 4 implies coma or vegetative state and CPC 5 stands for death. Neurological performance was assessed at 180 days after the CA.

## Statistical methods

Patients’ characteristics were compared using Student’s *t* test if normally distributed and expressed as mean ± standard deviation. The chi-square test and Fisher’s exact test (when expected frequency of five or less) were used to compare categorical values. Descriptive statistics were used for continuously measured SctO_2_ values and are expressed as mean ± standard deviation.

The data as collected are longitudinal in nature: SctO_2_ was measured repeatedly over time. By averaging SctO_2_ values per hours, the data yields 48 measurements per patient. To take the longitudinal nature of the data into account, a linear mixed model with a random intercept and a random slope was used [12].

The comparison of the evolution of SctO_2_ for the survivors versus non-survivors was of primary interest. To take possible confounders into account, an effect of gender and age, together with a quadratic effect of time, and all interactions with gender and survival were considered in a first, elaborated model.

A backward selection procedure was performed to exclude non-significant effects. This resulted in the following model:

$$ \begin{array}{l}Sa{t}_{ij}=\hfill \\ {}\left({\beta}_0^{ms}+{b}_{0i}\right)+\left({\beta}_1^{ms}+{b}_{1i}\right){t}_{ij}+{\beta}_2^s{t}_{ij}^2+{\beta}_3^{ms} ag{e}_i + {\beta}_4^m{t}_{ij} ag{e}_i+{\upepsilon}_{ij}\ \mathrm{Male}\ \mathrm{survivors}\hfill \\ {}\left({\beta}_0^{mn}+{b}_{0i}\right) + \left({\beta}_1^{mn}+{b}_{1i}\right){t}_{ij}+{\beta}_2^n{t}_{ij}^2+{\beta}_3^{mn} ag{e}_i+{\beta}_4^m{t}_{ij} ag{e}_i + {\upepsilon}_{ij}\ \mathrm{Male}\ \mathrm{n}\mathrm{o}\mathrm{n}\hbox{-} \mathrm{survivors}\hfill \\ {}\left({\beta}_0^{fs}+{b}_{0i}\right) + \left({\beta}_1^{fs}+{b}_{1i}\right){t}_{ij}+{\beta}_2^s{t}_{ij}^2+{\beta}_3^{fs} ag{e}_i+{\beta}_4^f{t}_{ij} ag{e}_i + {\upepsilon}_{ij}\ \mathrm{Female}\ \mathrm{survivors}\hfill \\ {}\left({\beta}_0^{fn}+{b}_{0i}\right) + \left({\beta}_1^{fn}+{b}_{1i}\right){t}_{ij}+{\beta}_2^n{t}_{ij}^2+{\beta}_3^{fn} ag{e}_i+{\beta}_4^f{t}_{ij} ag{e}_i + {\upepsilon}_{ij}\ \mathrm{Female}\ \mathrm{n}\mathrm{o}\mathrm{n}\hbox{-} \mathrm{survivors}\hfill \end{array} $$

Where *Sat*_*ij*_ and *t*_*ij*_ are SctO_2_measurement and the corresponding time of this measurement for patient *i* on hour *j*, *age*_*i*_ is the age of patient *i*, and $$ \left({b}_{0i},\;{b}_{1i}\right)\sim N\left(\left(\begin{array}{c}0\\ {}0\end{array}\right),\left(\begin{array}{cc}{d}_{11}& {d}_{12}\\ {}{d}_{12}& {d}_{22}\end{array}\right)\right) $$, are the random intercept and random slope respectively. All parameters in this model were significant, and no further reduction could be obtained. A *p* value < 0.05 was considered to be statistically significant. Tests were performed using IBM SPSS version 20.00 (IBM Corp., Armonk, NY, USA) and SAS Software version 13.2 (SAS Institute, Cary, NC, USA). Figures were made using GraphPad Prism 5.01 (GraphPad Software, San Diego, CA, USA).

## Results

**Fig. 1:**
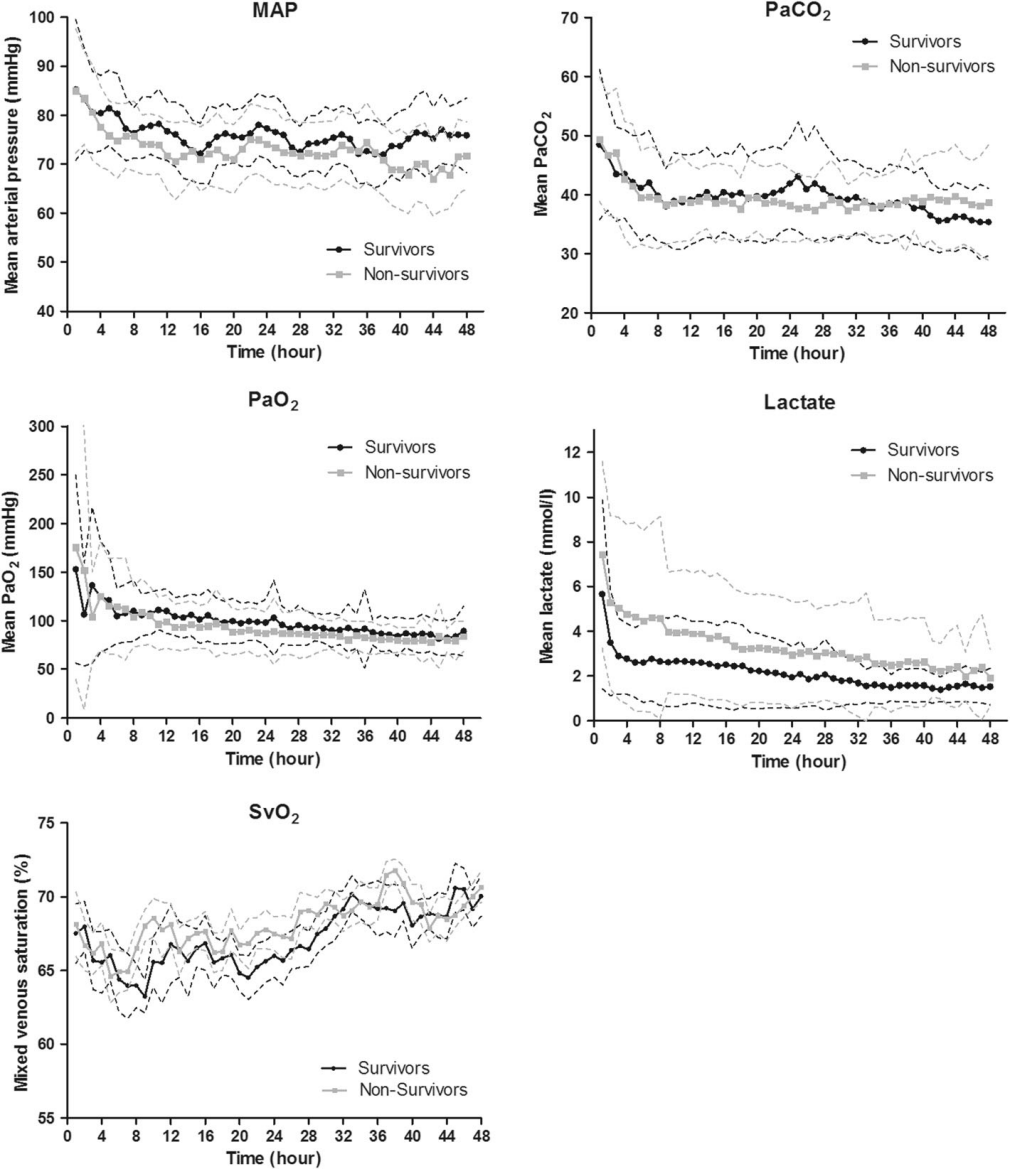
Course of hemodynamic parameters, mean arterial pressure, arterial carbon dioxide pressure, arterial oxygen pressure, lactate and mixed venous saturation. In this figure, the course for 48 hours of different hemodynamic parameters is shown as mean ± standard deviation. Overall *p* values: MAP: *p* = 0.020; PaCO_2_: *p* = 0.842; PaO_2_: *p* = 0.370; lactate: *p* = 0.002; SvO_2_: *p* = 0.649. *MAP* mean arterial pressure, *PaCO*_*2*_ arterial carbon dioxide tension, *PaO*_*2*_ arterial oxygen tension, *SvO*_*2*_ mixed venous saturation

One hundred and seven consecutive OHCA patients were included in this study. Baseline characteristics are summarized in Table 1. Fifty patients (47 %) survived with a good neurological outcome (CPC1–2) at 180 days post-CA. A significant difference in initial rhythm was observed, 84 % of the survivors had ventricular fibrillation in contrast to 41 % of the non-survivors (*p* < 0.001). Significantly more survivors underwent urgent coronary angiography (92 % versus 71 %, *p* = 0.008) and received a percutaneous coronary intervention (71 % versus 39 %, *p* = 0.001). We observed no significant difference in the use of IABP between both groups (*p* = 0.969). Within the group of survivors, 39 (78 %) patients had a CPC 1 and 11 (22 %) had a CPC 2 at 180 days post-CA. None of the survivors had a CPC 3 or CPC 4. Twelve patients died within 48 hours, with a mean age of 67 years ± 11, of whom four (33 %) were women. The mean SctO_2_ of these patients was 65 % ± 7. In Fig. 1 the course of the hourly mean MAP, arterial oxygen partial pressure (PaO_2_), arterial carbon dioxide partial pressure (PaCO_2_), SvO_2_ and lactate during the first 48 hours is shown for survivors and non-survivors.

**Table 1:** Patient demographics

	All patients	Survivors	Non-survivors	*p* value
Patients, n (%)	107	50 (47)	57 (53)	/
Age, mean (± SD)	63 (13)	61 (13)	65 (13)	0.084
Gender, _male/female, n (%)_	75 (70)/32 (30)	40 (78)/11 (22)	35 (63)/21 (37)	0.072
Witnessed, n (%)	91 (85)	46 (92)	45 (79)	0.182
Bystander BLS, n (%)	59 (55)	28 (55)	31 (55)	0.962
Initial rhythm				
VF_, n (%)_	66 (62)	42 (84)	24 (42)	<0.001
PEA_, n (%)_	11 (10)	4 (8)	7 (12)	0.374
Asystole_, n (%)_	25 (23)	4 (8)	21 (37)	<0.001
Time _emergency call – ROSC (min)_	30 ± 19	27 ± 17	34 ± 20	0.099
Cooling, _endovascular/surface, n (%)_	46 (43)/61(57)	26 (51)/25 (49)	20 (36)/36 (64)	0.111
Coronary angiography	87 (81)	46 (92)	41 (71)	0.008
PCI, n (%)	58 (54)	36 (72)	22 (39)	0.001
IABP, n (%)	25 (23)	12 (24)	13 (23)	0.969

*BLS* basic life support, *VF* ventricular fibrillation, *PEA* pulseless electrical activity, *ROSC* return of spontaneous circulation, *PCI* percutaneous coronary intervention, *IABP* intra-aortic balloon pump

**Fig. 2:**
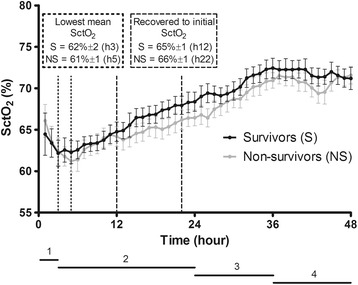
Cerebral saturation course (mean ± standard deviation). 1 = time to target temperature; 2 = therapeutic hypothermia; 3 = rewarming; 4 = normothermia. SctO_2_, cerebral tissue oxygen saturation

The mean SctO_2_ of the first hour after admission at the coronary care unit was 64 % ± 7 in survivors compared to 66 % ± 6 in non-survivors (*p* = 0.184). The mean SctO_2_ over 48 hours was significantly higher in the survivors (68 % ± 4) compared to non-survivors (66 % ± 5; *p* = 0.035). The mean course of SctO_2_ in both groups is given in Fig. 2. An initial decrease was observed after initiation of TTM until hour 3 in survivors (-5 % ± 6) and until hour 5 in non-survivors (-3 % ± 12; *p* = 0.432) followed by a progressive increase in both groups.

The first 48 hours of TTM at 33 °C after CA can be divided in four different phases: the cooling phase, followed by the hypothermia phase at 33 °C, the rewarming phase and finally the normothermia phase. The mean time to target temperature (cooling phase) was 183 min ± 160 in both groups. The hypothermia phase took 21 hours followed by a 12-hour rewarming phase and a 12-hour normothermia phase. The results of the mean SctO_2_ in each phase are listed in Table 2. We observed a significant difference in the mean SctO_2_ in the rewarming phase between survivors and non-survivors (70 % ± 1 versus 68 % ± 1, *p* = 0.046). No significant differences were observed in the other phases.

**Table 2:** Cerebral saturation values phase by phase

	Survivors	Non-survivors	*p* value
Cerebral saturation (%)			
Time to target temperature (0–3 h)	63 ± 2	64 ± 2	0.509
Therapeutic hypothermia (3–24 h)	65 ± 1	64 ± 1	0.076
Rewarming (24–36 h)	70 ± 1	68 ± 1	0.046
Normothermia (36–48 h)	72 ± 1	71 ± 1	0.217

If SctO_2_ values during the four different cooling phases were compared between patients with and without IABP, a difference was observed during the cooling and hypothermia phase with higher SctO_2_ values in the no-IABP group (*p* = 0.009 and *p* = 0.042). In the next two phases, the rewarming (phase 3) and normothermia phase (phase 4), no significant difference in SctO_2_ values was observed between both groups (phase 3 - *p* = 0.722; phase 4 - *p* = 0.827).

The area below a preset SctO_2_ threshold was calculated as well. Receiver operating curve (ROC) analysis revealed the highest area under the curve (AUC) for a SctO_2_ threshold of 55 % (AUC 0.58; specificity 52 % and sensitivity 62 %).

**Fig. 3:**
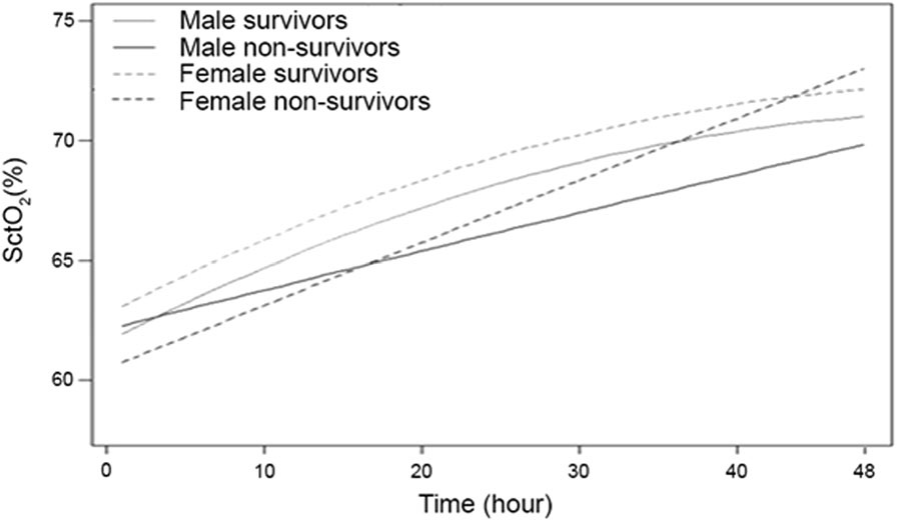
Fitted cerebral saturation by linear mixed models. SctO_2_, cerebral tissue oxygen saturation

If data were fitted in an optimal mixed model, a significant difference was observed between survivors and non-survivors concerning the course of SctO_2_ after adjustment for age and gender (*p* < 0.001) (Fig. 3). Between female survivors and non-survivors we observed a significant difference already from the start of TTM at 33 °C with a logarithmic course of SctO_2_ in the survivors group versus a linear one in the non-survivor group. Male survivors and non-survivors had initially similar SctO_2_ values but during induction of hypothermia, SctO_2_ increased more rapidly in the male survivors (logarithmic) compared to male non-survivors (linear).

## Discussion

In this study, SctO_2_ was prospectively monitored in OHCA patients with a presumed cardiac cause during the initial 48 hours after admission to the CCU. This is currently the largest post-resuscitation patient cohort evaluated using SctO_2_ to prognosticate outcome. In all patients TTM at 33 °C was applied for 24 hours, followed by an active rewarming at 0.3 °C/hour. Within this timeframe, we observed a significant different course of SctO_2_ between survivors (CPC 1–2) and non-survivors (CPC 5). In the rewarming phase, significant higher SctO_2_ values were observed in patients with a favorable neurological outcome. However, the clinical significant difference we observed in SctO_2_ course is of unlikely clinical meaning since this information is not available at the bedside, and moreover it is so small that at present clinicians will not be able to use any SctO_2_ cutoff value to predict outcome.

Experimental studies on CA and outcome revealed that the severity of brain damage is mainly influenced by the duration of the CA and by the mismatch in the oxygen extraction rate (CEO_2_) to CBF during the post-resuscitation period [13]. A better understanding of cerebral hemodynamic disturbances may have an impact on the post-CA management and may also allow a better prognostication. Monitoring of SctO_2_ could provide a non-invasive assessment of these cerebral hemodynamic disturbances.

In recent years, several studies investigated whether NIRS could be used during the post-CA stage to assist with the neuroprognostication and as a therapeutic target [6, 14, 15]. Overall, significant higher SctO_2_ were observed at different time points in the post-CA phase between patients with a favorable compared to unfavorable outcome. Nevertheless, the included patient populations were inhomogeneous (mix of OHCA and in-hospital cardiac arrest (IHCA) patients), rather small in sample size and mean SctO_2_ values over several hours were used.

In our patient cohort, we found the best AUC for a threshold value of 55 %. However, this AUC had a very low sensitivity and specificity. In contrast, Storm et al. found the highest AUC for outcome prediction at a SctO_2_ threshold of 50 % with a far higher sensitivity and specificity (AUC 0.80; specificity 70 % and sensitivity 86 %) [14]. We should remark that they included 60 both IH- and OHCA patients of which 38 % had a good neurological outcome compared to 47 % in our exclusively OHCA patient population. In the setting of aortic arch surgery, Fischer et al. showed that the time under SctO_2_ thresholds of 55 %, 60 %, and 65 % was associated with poor outcome [16]. In contrast to the perioperative setting where treatment algorithms are proposed to treat cerebral desaturations, no target SctO_2_ values are currently recommended in the post-CA phase.

In contrast to previous mentioned studies which applied an hour-by-hour analysis, we performed a linear mixed model analysis using continuous SctO_2_ data to investigate whether the SctO_2_ course over time was different between survivors versus non-survivors [6, 14, 15]. If all SctO_2_ values were fitted in a linear mixed model, a significant time effect was observed during the SctO_2_ course. More specifically, survivors followed a logarithmic SctO_2_ course over time compared to a more linear one for non-survivors. Based on these findings, it seems possible that the balance between oxygen supply and demand in survivors recovered more rapidly and that non-survivors have longer-lasting disturbances in cerebrovascular autoregulation. This implies that SctO_2_ in non-survivors could be more dependent on hemodynamic parameters such as MAP, CO, and PaCO_2_. Despite this significant discordance in time course, which probably indicates different underlying pathophysiologic mechanisms between both patient groups, we should take into account that this represents post hoc information, not available at the bedside. The SctO_2_ course of both, male and female patients with good favorable neurological outcome, follow the same shape compared to the patients with unfavorable outcome. However, the overall course of female survivors is higher compared to the male survivors, which is more or less also the case in the non-survivor group. Bickler et al. observed no difference in gender using the FORE-SIGHT technology to measure cerebral saturation in healthy volunteers [17]. In this way a bias by the used technology can be excluded. In healthy volunteers, regional cerebral blood flow is higher in female volunteers compared to male volunteers [18, 19]. In animal studies a greater cerebral cortical blood flow and lower cerebral oxygen extraction ratio was observed after severe cerebral hemorrhage and ventricular fibrillation in females [20]. However, these studies were performed without the use of TTM at 33 °C. All previous described findings might explain the higher observed SctO_2_ in female patients if we assume that TTM at 33 °C has a similar effect on both sexes. Another explanation could be the number of included female patients, which is only one third of the total included population.

At the initiation of TTM at 33 °C, a decrease in mean SctO_2_ was observed followed by a progressive increase in mean SctO_2_ in both survivors and non-survivors. This decrease could be explained by the onset of different pathophysiological mechanisms after CA. A period of delayed cerebral hypoperfusion occurs which is associated with an increase in cerebrovascular resistance, a drop in CBF, and cerebral metabolic oxygen consumption [21, 22]. Moreover, it has been shown that blood viscosity is higher during the initial hours after a CA [23–26]. As a significant negative correlation exists between blood viscosity and the mean flow velocity of the middle cerebral artery, this could explain the observed decrease in SctO_2_ [26]. Third, hemodynamic parameters such as PaCO_2_, MAP, and CO influence SctO_2_ [27]. Especially our observed decrease in PaCO_2_ (50 mmHg until 40 mmHg) with its subsequent effects on cerebral vasculature and CBF could influence SctO_2_ values [28]. Additionally, we observed a simultaneous decrease in MAP until 5–6 hours after the induction of TTM at 33 °C together with a decrease in SctO_2_ suggesting an impaired autoregulation. Cerebrovascular autoregulation is known to be disturbed or right shifted after a CA, which can influence SctO_2_ [21, 29]. Finally, CO, in the initial phase after a CA, is relatively low due to myocardial dysfunction [30]. We observed a decrease in SvO_2_, and as SvO_2_ is highly correlated with CO, our observed decrease in SctO_2_ can therefore be partially explained by a drop in CO [22, 30–32]. The hemodynamic variability in the initial hours after CA as described above, suggest the presence of a therapeutic window. Therefore, hemodynamic parameters such as MAP and CO could be optimized using an interventional protocol, which may prevent the potential harming cerebral desaturation in the early post-CA hours.

After the initial drop in mean SctO_2_, a progressive increase in mean SctO_2_ was observed reaching stable values around hour 12 and 22 in survivors and non-survivors, respectively. This 10-hour delay in SctO_2_recovery in non-survivors may implicate once more that brain recovery from ischemia after CA is not similar in survivors versus non-survivors. Since others described a low CEO_2_ together with a gradual increase in mean flow velocity until 48 hours after CA, our observed increase in SctO_2_ 6 hours after the start of TTM at 33 °C is supported by these findings [13, 33]. In addition, TTM at 33 °C induces a leftward shift of the oxygen dissociation curve. This results in an enhanced affinity of oxygen to hemoglobin, a phenomenon which may explain the progressive increase in SctO_2_ as well.

During the rewarming phase, using a rewarming rate of 0.3 °C/hour, we observed significant higher SctO_2_ values in survivors compared to non-survivors (*p* = 0.046). This finding has probably an influence on the statistical difference we observed in the overall course of SctO_2_. The optimal rewarming rate in post-CA patients (after TTM at 33 °C) is not known thus far. But both animal and human studies performed during cardiac surgery suggest a detrimental effect of rapid rewarming at the expense of potential neuroprotective effects of therapeutic hypothermia (TH) [34–37]. Therefore, our results can only indicate that rewarming has a different influence on cerebral hemodynamics in survivors versus non-survivors.

If a sub-analysis is performed comparing patients with and without an IABP, significant higher SctO_2_ values are observed during cooling and hypothermia in the no-IABP group. This represents probably the higher hemodynamic instability of patients receiving an IABP.

Our study has several limitations. First, we did not assess cerebral hemodynamic parameters by transcranial Doppler. The continuous measurement of both SctO_2_ and cerebral hemodynamic parameters could have allowed a better understanding of cerebral hemodynamic changes in post-CA patients. Second, SctO_2_ was measured using NIRS technology on the forehead. This is a regional measurement with the disadvantage that we do not have any information other than the frontal region. The number of patients included was rather limited but as far as we know, this study is currently the largest in which SctO_2_ is prospectively measured in post-CA patients. Nevertheless, we suppose that extending the number of patients will be of no added value for a better understanding of the underlying pathophysiologic mechanism responsible for the observed SctO_2_ course. For this purpose, an experimental setting using non-invasive as well as invasive cerebral hemodynamic measurements could provide more valuable information. At last, in this study SctO_2_ was measured during TTM at 33 °C. Consequently, these findings might not be applicable to patients treated with TTM at 36 °C. More importantly, significant higher SctO_2_ values were measured in the favorable neurological outcome group during the rewarming phase, which is absent in post-CA patients treated with TTM at 36 °C.

## Conclusion

This study represents the largest post-resuscitation cohort evaluated using NIRS technology, including a sizeable cohort of balloon-assisted patients. Although a significant difference was observed in the overall course of SctO_2_ between OHCA patients with a favorable and unfavorable outcome, the margin was too small to likely represent functional outcome differentiation based on SctO_2_ alone. As such, these results given such methodology as performed in this study suggest that NIRS is insufficient by itself to serve in outcome prognostication, but there may remain benefit when incorporated into a multi-neuromonitoring bedside assessment algorithm.

## Abbreviations

AUC: area under the curve
CA: cardiac arrest
CBF: cerebral blood flow
CCU: coronary care unit
CEO_2_: oxygen extraction rate
CO: cardiac output
CPC: cerebral performance category
CT: computed tomography
IABP: intra-aortic balloon pump
IHCA: in-hospital cardiac arrest
MAP: mean arterial pressure
NIRS: near-infrared spectroscopy
OHCA: out-of-hospital cardiac arrest
PaCO_2_: arterial carbon dioxide partial pressure
PaO_2_: arterial oxygen partial pressure
ROC curve: receiver operating characteristics curve
ROSC: return of spontaneous circulation
SctO_2_: cerebral tissue oxygen saturation
SvO_2_: mixed venous blood oxygen saturation
TH: therapeutic hypothermia
TTM: targeted temperature management
